# Distal posterior inferior cerebellar artery dissecting aneurysms: a systematic review and meta-analysis

**DOI:** 10.1007/s00701-025-06603-7

**Published:** 2025-07-26

**Authors:** Mustafa Ismail, Norito Kinjo, Rania H. Al-Taie, Imad Samman Tahhan, Hasna Loulida, Alejandro M. Spiotta

**Affiliations:** 1 Department of Neurosurgery, Medical University of South Carolina, Charleston, SC 29425 USA; 2 Department of Surgery, College of Medicine, Mustansiriyah University, Baghdad, Iraq

**Keywords:** Deconstructive techniques, Dissection, Posterior inferior cerebellar artery, Pseudoaneurysm, Reconstructive techniques

## Abstract

**Background:**

Distal posterior inferior cerebellar artery (PICA) dissecting aneurysms are rare, anatomically complex, and clinically underreported. Management strategies remain poorly standardized, and long-term outcomes are not well defined. This paper aims to systematically review the anatomical, clinical, and therapeutic characteristics of distal PICA dissecting aneurysms.

**Methods:**

Following PRISMA guidelines, a comprehensive search of PubMed and SCOPUS identified 15 eligible studies, including 68 patients. Data on clinical presentation, aneurysm characteristics, treatment strategies, and outcomes were extracted. Pooled proportions and meta-analyses were conducted using random-effects models.

**Results:**

Among 68 patients, 91.2% presented with subarachnoid hemorrhage. Endovascular treatment was preferred (69.1%), achieving a higher complete occlusion rate (90%) and low retreatment (9%), and rebleeding rates (8%). Surgical treatment (23.5%) had similar efficacy (81% occlusion). The complete occlusion rate was not significantly different between the two (OR: 1.43; log OR = 0.360; 95% CI: –0.848 to 1.569, p = 0.559). Functional outcomes were favorable overall, with 79.4% of patients achieving mRS < 2. Stroke was the most frequent complication (14.2%). No significant differences were found between reconstructive and deconstructive approaches (p = 0.971). Flow diversion was not utilized in any case.

**Conclusion:**

Distal PICA dissecting aneurysms, though uncommon, pose a high risk of hemorrhage. Endovascular parent artery occlusion offers high occlusion rates with low morbidity. The absence of flow-diversion strategies highlights current device limitations and illustrates the need for anatomy-specific, prospective studies to guide optimal management.

## Introduction

Aneurysms of the distal posterior inferior cerebellar artery (PICA) are uncommon, accounting for about 1.4% to 4.5% of all intracranial aneurysms [17]. Within this category, dissecting aneurysms are even rarer, making up only 0.5% to 0.7% of cerebral aneurysms [15]. Their infrequency and potential underreporting, particularly among smaller or asymptomatic cases, make it difficult to determine the actual incidence. Typically, these aneurysms manifest with abrupt and severe symptoms such as neck pain, ischemic deficits, or subarachnoid hemorrhage (SAH), particularly when the dissection penetrates all layers of the vessel wall [2, 15].

Understanding the clinical relevance of these aneurysms requires appreciating the complex and variable anatomy of PICA. The artery supplies vital posterior fossa structures, including the inferior vermis, tonsils, cerebellar hemispheres, medulla, choroid plexus, and tela choroidea of the fourth ventricle [17]. The fourth ventricle's choroid plexus receives its main supply from the PICA, which provides most branching vessels to this area [23]. This distribution highlights the potential for significant neurological compromise with any intervention that risks disrupting PICA flow.

Management of dissecting PICA aneurysms has historically relied on institutional and surgeon preferences rather than standardized protocols. Surgical options like clipping, wrapping, or trapping, with or without bypass, have shown variable success [12]. Recently, endovascular approaches have become appealing as they reduce the manipulation of critical neurovascular structures in the brainstem and lower cranial nerve regions [3, 6]. Parent artery occlusion (PAO) is the most common endovascular technique, but it carries risks in areas with weak collateral supply. Some authors suggest coil embolization of the aneurysm sac while preserving the parent artery to maintain flow and lower PICA territory infarction [3, 6]. Nonetheless, current literature consists mainly of isolated case reports and small series, often failing to differentiate between proximal and distal PICA aneurysms. Therefore, optimal management of distal dissecting and pseudoaneurysmal lesions of the PICA remains poorly defined.

The present review aims to consolidate current knowledge on PICA dissecting and pseudoaneurysms, focusing on anatomical nuances, treatment paradigms, and clinical outcomes.

## Methods

### Search strategy and study selection

This systematic review was conducted following the PRISMA (Preferred Reporting Items for Systematic Reviews and Meta-Analyses) guidelines [22]. A comprehensive literature search was performed across two major scientific databases, PubMed and SCOPUS, using the Boolean search string: (Dissecting OR pseudoaneurysm) AND (Distal (PICA OR posterior inferior cerebellar artery)).

Eligible studies included case reports, case series, and observational studies that reported on distal PICA dissecting or pseudoaneurysmal lesions, with extractable data on clinical presentation, radiological findings, treatment modalities, and patient outcomes. Exclusion criteria included non-English articles, conference abstracts without full texts, review articles, and studies with insufficient clinical data.

Rayyan, a web-based systematic review platform, was used for blinded and independent screening of titles, abstracts, and full texts by two reviewers. Disagreements were resolved through consensus.

### Data extraction and quality assessment

Data extraction was performed independently by two authors using a structured form and included the following parameters: study details, demographics, aneurysm characteristics, clinical symptoms, radiographic findings, modality of treatment (surgical vs. endovascular), use of bypass or adjunctive techniques, and outcome measures such as mortality, rebleeding, complications, and functional recovery (mRS scores). Risk of bias in included case reports was assessed using the CARE checklist, while the ROBINS-I tool was applied to case series [1, 25]. Studies were classified as having low, moderate, or serious risk of bias accordingly.

### Statistical analysis

Descriptive statistics were calculated to summarize baseline characteristics and treatment patterns. A one-arm proportional meta-analysis was conducted to pool frequencies of key outcomes such as PICA patency preservation, long-term occlusion success, and mortality. Where comparative data were available, dichotomous meta-analyses were also performed to evaluate treatment differences between surgical and endovascular interventions.

All analyses were performed using Jamovi (version 2.3) with random-effects models applied due to anticipated heterogeneity. P value < 0.05 is considered statistically significant. Heterogeneity was assessed using the I^2^ statistic, and results were visualized using forest plots where appropriate.

## Results

**Fig. 1:**
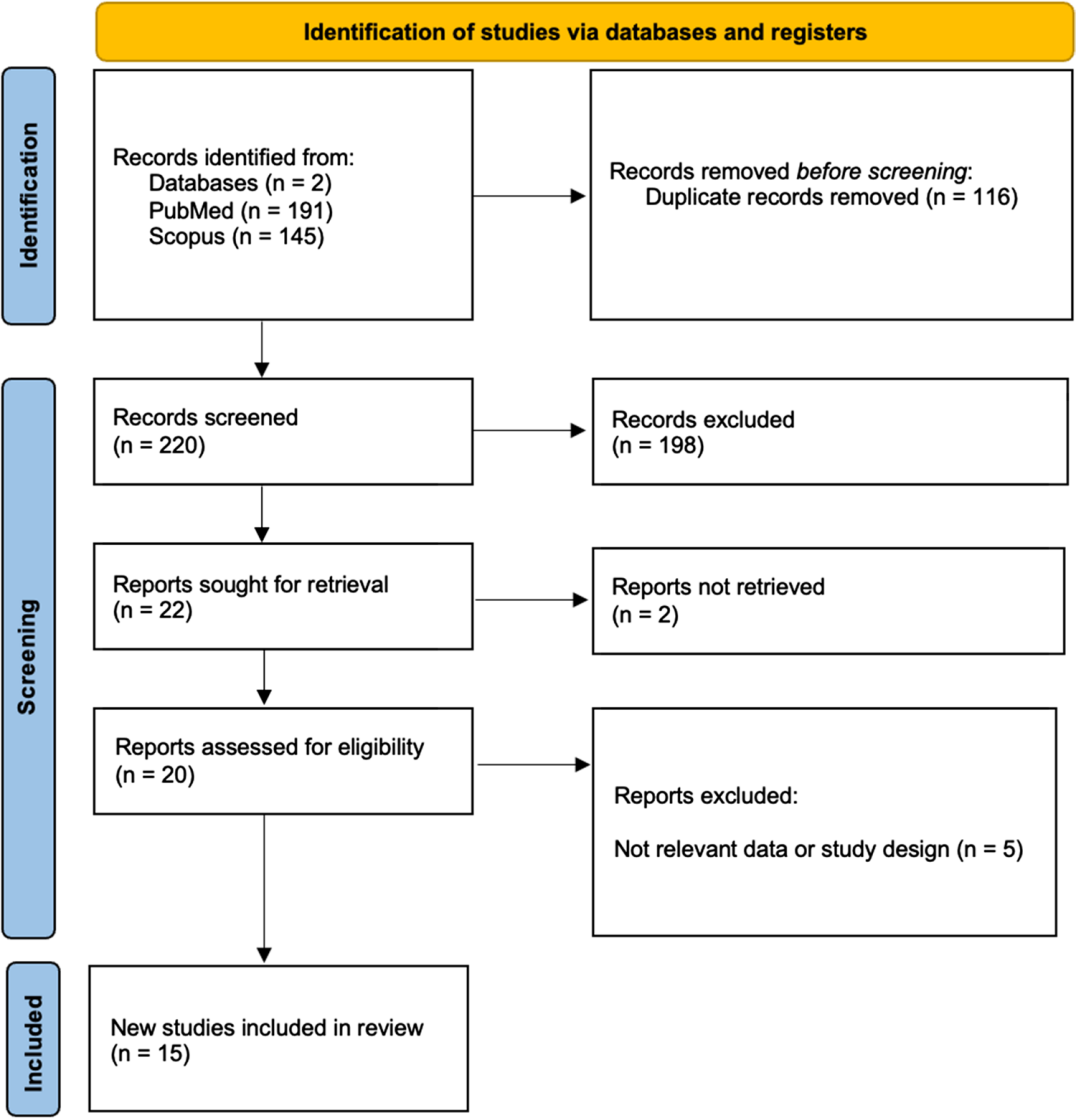
PRISMA flowchart of the included studies

A total of 336 records were initially identified, 145 from SCOPUS and 191 from PubMed. After removing duplicates, 220 unique articles remained for screening. Following title, abstract, and full-text review based on eligibility criteria, 15 studies were ultimately included in the final analysis (Fig. 1).

### Study characteristics and demographics

A total of 15 studies published between 1991 and 2024 were included in this review, encompassing 68 patients (Table 1). Out of the 68 patients, 30 (44.1%) were male. Only one study mentioned racial or ethnic information, reporting a case involving an African American patient. Notably, 62 out of 68 patients (91.2%) presented with ruptured aneurysms. Comorbidity data were variably reported across studies. Hypertension was the most common comorbidity, present in at least 20 patients (29.4%). Other noted conditions included diabetes mellitus, inflammatory bowel disease, prior subarachnoid hemorrhage, and malignancy.

**Table 1:** Baseline characteristics of included studies and patient demographics

Study ID	Author(s)	Year	Country	Study Design	N	Mean Age ± (Years)	No of Males	% of Males	Ethnicity (N, %)	Race (N, %)	Family History	Co-morbidities	Ruptured vs. Unruptured (N, %)	Mean Follow-up ± SD (Months)
1	Yamaura et al. [7]	1991	Japan	Case series	3	58.0 ± 8.60	1	33.3%	NR	NR	NR	HTN (2), DM (1)	2, 66.7%	28.0 ± 24.25
2	Jafar et al. [10]	1998	USA	Case report	1	48 ± 0.0	1	100%	NR	NR	NR	HTN	1, 100% Ruptured	6.0 ± 0.0
3	Taylor et al. [18]	2001	Canada	Case report	1	49 ± 0.0	0	0%	NR	NR	NR	HTN and IBD that Salazopyrine controlled	1, 100% Ruptured	NR
4	Yamakawa et al. [20]	2004	Japan	Case report	2	52 ± 3	1	50%	NR	NR	NR	HTN (both)	2, 100% Ruptured	NR
5	Nussbaum et al. [19]	2008	USA	Case series	6	49.2 ± 10.0	4	66.7%	NR	NR	NR	NR	5 Ruptured, 1 Unruptured	21.6 (6–48)
6	Lim et al. [15]	2010	Korea	Case series	6	53.3 ± 17.9	2	33.3%	NR	NR	NR	NR	5 Ruptured, 1 Unruptured	20.5 (3–69)
7	Ioannidis et al. [9]	2012	Greece	Case series	10	58.3 ± 6.7	3	30%	NR	NR	NR	NR	10, 100% Ruptured	12 (6–28)
8	Puri et al. [21]	2014	USA	Case series	7	69.3 ± 11.8	4	57.1%	NR	NR	NR	NR	7, 100% Ruptured	5.8 (0.75–10)
9	Li et al. [14]	2015	China	Case series	17	53.5 ± 10.8	8	47.1%	NR	NR	NR	HTN (5), Hydrocephalus (5)	17 Ruptured	13.5 (3–30)
10	Chung et al. [4]	2016	Korea	Case series	10	60.8 ± 14.5	3	30%	NR	NR	NR	HTN (5), DM (5), trauma (1)	7 Ruptured, 3 Infarction	29.2 (12–52)
11	Lee et al. [13]	2016	Korea	Case report	1	55 ± 0.0	0	0%	NR	NR	NR	NR	1, 100% Ruptured	6 ± 0.0
12	Khattar et al. [11]	2020	USA	Case report	1	57 ± 0.0	0	0%	NR	African American	NR	HTN, prior breast cancer	1, 100% Ruptured	3 ± 0.0
13	Lim et al. [16]	2022	Korea	Case report	1	66 ± 0.0	1	100%	NR	NR	NR	HTN, DM, Angina	1, 100% Ruptured	8 ± 0.0
14	Shintoku et al. [24]	2023	Japan	Case report	1	68 ± 0.0	1	100%	NR	NR	NR	HTN, DM, prior SAH	1, 100% Ruptured	12 ± 0.0
15	Hiratsuka et al. [8]	2024	Japan	Case report	1	41 ± 0.0	1	100%	NR	NR	NR	HTN	1, 100% Ruptured	7 ± 0.0

The most frequently reported symptom was headache, observed in 22 patients (32.4%), highlighting the brainstem and cerebellar involvement typical of distal PICA pathology. Loss of consciousness (LOC) was documented in 3 patients (4.4%), and coma at presentation occurred in 2 patients (2.9%), typically in the setting of subarachnoid hemorrhage. Cranial nerve palsy was rare, seen in 1 patient (1.5%), including one case of isolated sixth nerve (abducent) palsy. Additional neurological signs included photophobia and meningismus (reported in one case), and neck stiffness in another, both pointing toward meningeal irritation from hemorrhage. Notably, seizures and visual disturbances were not reported in any included study, suggesting a relatively localized effect of the aneurysmal pathology on the posterior fossa.

### Diagnosis and initial management

The mean time to diagnosis varied widely across studies, ranging from immediate recognition on initial imaging to delays of up to 30 days, particularly in post-traumatic or atypical presentations (Table 2). The hallmark imaging findings across studies included fusiform dilations and double lumens, indicative of arterial dissection. In rare instances, spinal hematomas and venous stasis were also documented, underscoring the anatomical variability of the disease.

**Table 2:** Diagnostic modalities, radiographic findings, and management strategies

Author(s)	Mean Time to Diagnosis ± SD (Days)	Diagnostic Modality (N, %)	Imaging Findings	Medical Management (N, %)	Endovascular Intervention (N, %)	Surgical Intervention (N, %)	Bypass Used (N, %)	Other Techniques (e.g., Trapping, Occlusion) (N, %)
Yamaura et al. [7]	3.0 ± 3.0	Angiography (3, 100%)	Fusiform dilation, vessel narrowing, venous stasis, double lumen in some	0 (0%)	0 (0%)	3 (100%)	0 (0%)	Wrapping, trapping, intramural clot resection, shunt in 1
Jafar et al. [10]	Immediate	CT, Angio	Fusiform dissecting aneurysm of PICA with dynamic enlargement	0 (0%)	0 (0%)	1 (100%)	0 (0%)	Sundt clip + muslin wrap with flow preservation
Taylor et al. [18]	Same Day	CT, DSA	Fusiform dilation of proximal PICA	0 (0%)	0 (0%)	1 (100%)	0 (0%)	0 (0%)
Yamakawa et al. [20]	NR	DSA, CT	Fusiform/saccular dilations of distal PICA, ischemic changes	0 (0%)	0 (0%)	2 (100%)	1 (50%)	Trapping, resection, one end-to-end anastomosis
Nussbaum et al. [19]	Immediate	DSA, MRI	Fusiform dilations, blue-black vessel appearance, medullary involvement	0 (0%)	0 (0%)	6 (100%)	3 (50%)	Wrapping, clip reconstruction, shunting in some
Lim et al. [15]	Mean 3.5 days (1–10)	DSA + 3D angio	Fusiform dissecting aneurysms, distal segments (medullary–tonsillomedullary)	0 (0%)	5 (83.3%)	1 (16.7%)	1 (16.7%)	Coiling ± PVO; one surgical resection with reanastomosis
Ioannidis et al. [9]	Mean 8 days (1–30)	DSA (100%)	Fusiform dissecting aneurysms in anterior/lateral medullary, cortical PICA	0 (0%)	10 (100%)	0 (0%)	0 (0%)	NR
Puri et al. [21]	Immediate	DSA, CT	Distal dissecting aneurysms; SAH + IVH common	0 (0%)	7 (100%)	0 (0%)	0 (0%)	NR
Li et al. [14]	Mean 10.2 days (3–24)	DSA, CTA, MRA	Dissecting aneurysms (segments I–III); frequent SAH, IVH, hydrocephalus	0 (0%)	17 (100%)	0 (0%)	0 (0%)	NR
Chung et al. [4]	Within 1 week	DSA, CTA, MRA	Dissections mostly in segments I–II; pseudoaneurysm, double lumen, SAH or infarcts	3 (30%)	4 (40%)	3 (30%)	2 (20%)	Surgical trapping, bypass; one failed conservative case
Lee et al. [13]	30 days (delayed Dx)	CT, MRI, DSA	Traumatic pseudoaneurysm in cortical PICA (distal), post-blunt trauma	0 (0%)	0 (0%)	1 (100%)	0 (0%)	NR
Khattar et al. [11]	Immediate	CT, CTA, DSA	Dissecting aneurysm in caudal loop of PICA; SAH (Fisher 4); distal narrowing	0 (0%)	1 (100%)	0 (0%)	0 (0%)	Intrasaccular WEB
Lim et al. [16]	Immediate	CT, CTA, DSA	Long segment PICA dissection, “pearl and string”; SAH + IVH	0 (0%)	1 (100%)	0 (0%)	0 (0%)	NR
Shintoku et al. [24]	~ 1 week post-onset	CT, MRI, DSA	Cortical PICA dissection (vermian branch); irregular wall; collateral flow visible	0 (0%)	1 (100%)	0 (0%)	0 (0%)	Internal trapping via NBCA using DeFrictor nano catheter
Hiratsuka et al. [8]	Delayed (after spinal bleed)	MRI, CTA, T2*, DSA	Distal PICA dissecting aneurysm; spinal hematoma; double-origin PICA; SAH confirmed late	0 (0%)	1 (100%)	0 (0%)	0 (0%)	Parent vessel coiling; preserved flow via alternate PICA origin

Surgical intervention was the main approach for 16 patients (23.5%), especially in earlier studies or in cases involving mass effect, hydrocephalus, or unsuccessful endovascular access. Techniques used included trapping, resection, and clipping, occasionally enhanced by bypass procedures like end-to-end anastomosis or occipital artery-PICA bypass. Importantly, 7 patients underwent bypass, making up 10.3% of the entire cohort. Endovascular treatment was administered to 47 patients (69.1%), indicating an increasing trend toward less invasive methods in recent years (Table 3).

**Table 3:** Technical aspects of endovascular interventions

Author(s)	Access Location (N, %)	Coil (N, %)	Balloon (N, %)	Two Catheter Technique (N, %)	Stent (N, %)	Flow Diversion (N, %)	Any Other Type of Treatment (N, %)
Yamaura et al. [7]	NR	0 (0%)	0 (0%)	0 (0%)	0 (0%)	0 (0%)	Shunt placement (3, 100%)
Jafar et al. [10]	Not applicable (surgical)	0 (0%)	0 (0%)	0 (0%)	0 (0%)	0 (0%)	None (microsurgical clipping only)
Taylor et al. [18]	Not applicable (surgical)	0 (0%)	0 (0%)	0 (0%)	0 (0%)	0 (0%)	PICA to PICA anastomosis
Yamakawa et al. [20]	Not applicable (surgical)	0 (0%)	0 (0%)	0 (0%)	0 (0%)	0 (0%)	Trapping + end-to-end anastomosis (1); Trapping only (1)
Nussbaum et al. [19]	Not applicable (surgical)	0 (0%)	0 (0%)	0 (0%)	0 (0%)	0 (0%)	Wrap/clip (3); Prox occlusion + bypass (3); VP shunt (3/6 cases)
Lim et al. [15]	NR	5 (83.3%)	0 (0%)	0 (0%)	0 (0%)	0 (0%)	Surgical bypass (1); Re-treatment for recanalization (1)
Ioannidis et al. [9]	Femoral (10/10, 100%)	8 (80%)	10 (100%) (test)	0 (0%)	0 (0%)	0 (0%)	Glue embolization (2)
Puri et al. [21]	Femoral (7/7, 100%)	0 (0%)	4 (57.1%)	0 (0%)	0 (0%)	0 (0%)	Onyx embolization (7/7); Parent artery preservation (7/7)
Li et al. [14]	Femoral (17/17, 100%)	9 (52.9%)	0 (0%)	0 (0%)	1 (5.8%)	0 (0%)	PVO (7); Shunt (4); Re-treatment (1 with stent)
Chung et al. [4]	NR	2 (20%)	0 (0%)	0 (0%)	1 (10%)	0 (0%)	Triple stenting (1); Failed endovascular attempt (1)
Lee et al. [13]	Not applicable (surgical case)	0 (0%)	0 (0%)	0 (0%)	0 (0%)	0 (0%)	Cortical pseudoaneurysm resected via craniotomy
Khattar et al. [11]	Femoral (1/1, 100%)	0 (0%)	0 (0%)	0 (0%)	0 (0%)	0 (0%)	WEB device embolization (1/1); No recurrence; Vessel remodeling
Lim et al. [16]	Femoral (1/1, 100%)	0 (0%)	0 (0%)	0 (0%)	1 (100%)	0 (0%)	Overlapping LVIS Jr. stents (2); EVD pre-op, no shunt needed
Shintoku et al. [24]	Femoral (1/1, 100%)	0 (0%)	0 (0%)	0 (0%)	0 (0%)	0 (0%)	NBCA embolization (1); Wedged microcatheter technique
Hiratsuka et al. [8]	Femoral (1/1, 100%)	1 (100%)	0 (0%)	0 (0%)	0 (0%)	0 (0%)	Parent artery occlusion with coils; Lumbar drainage for ICP management

Coil embolization emerged as the most common endovascular technique, being performed in 25 patients, which accounts for 53.2% of all endovascular cases. This method was frequently combined with PVO strategies. Stent deployment was done in 3 instances. Importantly, flow diversion was absent from all included studies, likely due to limitations related to vessel caliber and the location of distal PICA. Additionally, a range of non-coiling embolic techniques was reported, including WEB device embolization (n = 1) and NBCA (glue) embolization (n = 3).

### Hemorrhagic patterns and clinical grading

Subarachnoid hemorrhage (SAH) was the most common radiological finding at presentation, documented in 59 patients (86.7%), affirming the hemorrhagic nature of distal PICA dissecting and pseudoaneurysmal lesions (Table 4). Intraventricular hemorrhage (IVH) was present in 32 patients (47.1%). Hydrocephalus at admission was observed in 10 patients (14.7%), most frequently in cases with extensive SAH or IVH. Notably, Nussbaum et al. reported hydrocephalus in 66.7% of their cohort, correlating with a high rate of intraventricular extension.

**Table 4:** Hemorrhagic findings and clinical severity scores in distal PICA lesions

Author(s)	Hunt and Hess Score ± SD	Fisher Score ± SD	SAH at presentation (N, %)	Subdural Hemorrhage (N, %)	Intraparenchymal Hemorrhage (N, %)	Intraventricular Hemorrhage (N, %)	Hydrocephalus at admission (N, %)
Yamaura et al. [7]	NR	NR	2/3 (66.67%)	0 (0%)	0 (0%)	0 (0%)	0 (0%)
Jafar et al. [10]	NR	NR	1/1 (100%)	0 (0%)	0 (0%)	1/1 (100%)	0 (0%)
Taylor et al. [18]	NR	NR	1/1 (100%)	0/1 (0%)	0/1 (0%)	0/1 (0%)	0/1 (0%)
Yamakawa et al. [20]	NR	NR	2/2 (100%)	0/2 (0%)	0/2 (0%)	2/2 (100%)	0/2 (0%)
Nussbaum et al. [19]	NR	NR	5/6 (83.3%)	0/6 (0%)	0/6 (0%)	3/6 (50%)	4/6 (66.67%)
Lim et al. [15]	2.75 ± 1.7	NR	4/6 (67%)	1/6 (17%)	1/6 (17%)	3/6 (50%)	0 (0%)
Ioannidis et al. [9]	2.3 ± 1.1	NR	10/10 (100%)	0/10 (0%)	1/10 (10%)	5/10 (50%)	0 (0%)
Puri et al. [21]	4.3 ± 0.5	NR	7/7 (100%)	0/7 (0%)	0/7 (0%)	0/7 (0%)	0 (0%)
Li et al. [14]	2.4 ± 0.9	NR	17/17 (100%)	0/17 (0%)	0/17 (0%)	9/17 (53%)	5/17 (29%)
Chung et al. [4]	NR	NR	7/10 (70%)	0/10 (0%)	0/10 (0%)	7/10 (70%)	0 (0%)
Lee et al. [13]	NR	NR	1/1 (100%)	1/1 (100%)	1/1 (100%)	0/1 (0%)	0/1 (0%)
Khattar et al. [11]	NR	4 (Modified Fisher)	1/1 (100%)	0/1 (0%)	0/1 (0%)	1/1 (100%)	0/1 (0%)
Lim et al. [16]	NR	NR	1/1 (100%)	0/1 (0%)	0/1 (0%)	1/1 (100%)	1/1 (100%)
Shintoku et al. [24]	NR	NR	1/1 (100%)	0/1 (0%)	0/1 (0%)	0/1 (0%)	0/1 (0%)
Hiratsuka et al. [8]	NR	NR	1/1 (100%)	1/1 (100%)	0/1 (0%)	0/1 (0%)	0/1 (0%)

### Aneurysm characteristics

Left-sided aneurysms were slightly more common, reported in 37 cases (54.4%), compared to right-sided, which occurred in 31 cases (45.6%). No bilateral lesions were reported. Thrombosis within the aneurysm sac was noted in 28 cases (41.2%). Most aneurysms were spontaneous, comprising 65 out of 68 cases (95.6%). Traumatic dissections accounted for 2 cases (2.9%), and one patient had a rare segmental mediolytic aneurysm. Size measurements varied; mean aneurysm width ranged from ~ 3 mm to over 10 mm, with the largest reaching 15 mm. Associated vascular pathologies were uncommon. Four patients in Li et al.’s series had coexisting intracranial aneurysms, while Khattar et al. documented a case with a dural arteriovenous fistula. Yamaura et al. also reported an additional vertebral artery aneurysm in one case (Table 5).

**Table 5:** Aneurysm morphology, etiology, and associated vascular pathologies

Author(s)	Aneurysm Side (N, %)	Thrombosed Aneurysm? (N, %)	Aneurysm Etiology (N, %)	Mean Width (mm) ± SD	Mean Height (mm) ± SD	Associated Vascular Pathology (N, %)	Same Territory Pathology
Yamaura et al. [7]	Left: 3 (100%)	Yes: 1 (17%)	Spontaneous: 3 (100%)	NR	NR	Another small aneurysm of the vertebral artery in one case	Yes
Jafar et al. [10]	Right: 1 (100%)	No: 0	Spontaneous: 1 (100%)	NR	NR	0	0
Taylor et al. [18]	Left: 1 (100%)	Yes: 1 (100%)	Spontaneous: 1 (100%)	1.8 mm	2.6 mm	0	0
Yamakawa et al. [20]	Case 1: Right Case 2: Left	Yes: 2/2 (100%)	Segmental mediolytic (1), Spontaneous (1)	NR	NR	0	0
Nussbaum et al. [19]	Left: 4 (67%) Right: 2 (33%)	NR	Spontaneous: 6 (100%)	< 10 mm all	< 10 mm all	0	0
Lim et al. [15]	Left: 4 (67%) Right: 2 (33%)	Yes: 6/6 (100%)	Spontaneous: 5 (83.3%) Traumatic: 1 (16.7%)	4.5 mm (range 2–12 mm)	NR	0	0
Ioannidis et al. [9]	Left: 4 (40%) Right: 6 (60%)	NR	Spontaneous: 10 (100%)	3–15 mm	3–15 mm	0	0
Puri et al. [21]	Left: 4 (57%) Right: 3 (43%)	Yes: 7/7 (100%)	Spontaneous: 7 (100%)	~ 3 mm (2.2–3.8 mm)	~ 2.5–3 mm	0	0
Li et al. [14]	Left: 10 (59%) Right: 7 (41%)	NR	Spontaneous: 17 (100%)	Mean: 5.2 mm	NR	Coexisting aneurysms (4, 23.5%)	NR
Chung et al. [4]	Left: 5 (50%) Right: 5 (50%)	Yes: 7/10 (70%)	Spontaneous: 10 (100%)	NR	NR	0	0
Lee et al. [13]	Right: 1 (100%)	Yes: 1/1 (100%)	Traumatic: 1 (100%)	4 mm	NR	0	0
Khattar et al. [11]	Right: 1 (100%)	Yes: 1/1 (100%)	Spontaneous: 1 (100%)	5 mm	NR	Dural arteriovenous fistula	Yes
Lim et al. [16]	Right: 1 (100%)	Yes: 1/1 (100%)	Spontaneous: 1 (100%)	~ 10 mm	NR	0	0
Shintoku et al. [24]	Left: 1 (100%)	NR	Spontaneous: 1 (100%)	NR	NR	0	0
Hiratsuka et al. [8]	Right: 1 (100%)	Yes: 1/1 (100%)	Spontaneous: 1 (100%)	NR	NR	0	0

### Perioperative management and complications

External ventricular drain (EVD) placement was performed in 15 patients (22.1%), primarily in cases of hydrocephalus or increased intracranial pressure associated with subarachnoid or intraventricular hemorrhage (Table 6). Lumbar drain (LD) placement was extremely rare, used in only 1 patient (1.5%) in the study by Hiratsuka et al. [], who presented with spinal subarachnoid extension and elevated intracranial pressure. No other studies reported LD usage during treatment. There were no cases of radiographic or symptomatic vasospasm documented across all 15 studies.

**Table 6:** Perioperative management and complications

Author(s)	Was EVD placed during admission (N, %)	EVD Placement (before or after) aneurysm treatment (N, %)	Was LD placed during admission (N, %)	Radiographic Vasospasm (N, %)	Symptomatic Vasospasm (N, %)	Access Site Complication (N, %)
Yamaura et al. [7]	0 (0%)	3 (100%)	0 (0%)	0 (0%)	0 (0%)	0 (0%)
Jafar et al. [10]	1 (100%)	Before (100%)	0 (0%)	0 (0%)	0 (0%)	0 (0%)
Taylor et al. [18]	1 (100%)	Before (100%)	0 (0%)	0 (0%)	0 (0%)	0 (0%)
Yamakawa et al. [20]	2 (100%)	Before (100%)	0 (0%)	0 (0%)	0 (0%)	0 (0%)
Nussbaum et al. [19]	4 (66.7%)	Before (100%)	0 (0%)	0 (0%)	0 (0%)	1 (16.7%) (Pneumonia/trach)
Lim et al. [15]	0 (0%)	0 (0%)	0 (0%)	0 (0%)	0 (0%)	0 (0%)
Ioannidis et al. [9]	0 (0%)	0 (0%)	0 (0%)	0 (0%)	0 (0%)	0 (0%)
Puri et al. [21]	0 (0%)	0 (0%)	0 (0%)	0 (0%)	0 (0%)	0 (0%)
Li et al. [14]	5 (29.4%)	Before (100%)	0 (0%)	0 (0%)	0 (0%)	1 (5.9%) (Intraop bleed)
Chung et al. [4]	0 (0%)	0 (0%)	0 (0%)	0 (0%)	0 (0%)	0 (0%)
Lee et al. [13]	1 (100%)	Before (100%)	0 (0%)	0 (0%)	0 (0%)	0 (0%)
Khattar et al. [11]	0 (0%)	0 (0%)	0 (0%)	0 (0%)	0 (0%)	0 (0%)
Lim et al. [16]	1 (100%)	Before (100%)	0 (0%)	0 (0%)	0 (0%)	0 (0%)
Shintoku et al. [24]	0 (0%)	0 (0%)	0 (0%)	0 (0%)	0 (0%)	0 (0%)
Hiratsuka et al. [8]	0 (0%)	0 (0%)	1 (100%)	0 (0%)	0 (0%)	0 (0%)

### Post-treatment outcomes and long-term prognosis

Postoperative complications were reported in 24 patients (35.3%), with the most common being asymptomatic infarcts and recurrence (Table 7). Isolated cases of cranial nerve palsy, mild leg weakness, and intracranial hypertension were also noted but were managed conservatively.

**Table 7:** Postoperative complications, functional outcomes, and long-term occlusion success

Author(s)	Pre-op Complications (N, %)	Post-op Complications	Preserved PICA Patency (N, %)	Mortality Rate (%)	Rebleeding Rate (%)	mRS Pre < 2 (N, %)	mRS Post < 2 (N, %)	Recanalization Rate (%)	100% Long-Term Occlusion Success (%)
Yamaura et al. [7]	0 (0%)	0 (0%)	3 (100%)	0%	0%	NR	3 (100%)	3 (100%)	2 (66.67%)
Jafar et al. [10]	0 (0%)	0 (0%)	1 (100%)	0%	0%	1/1 (100%)	1/1 (100%)	NR	1 (100%)
Taylor et al. [18]	0 (0%)	0 (0%)	1/1 (100%)	0%	0%	NR	NR	NR	1 (100%)
Yamakawa et al. [20]	0 (0%)	Case 1: None; Case 2: death post-op	1/2 (50%)	1/2 (50%)	0%	Case 1: Yes; Case 2: No	Case 1: Recovery; Case 2: Died	0%	2 (100%)
Nussbaum et al. [19]	0 (0%)	Tracheostomy (1), infarcts (2), hydrocephalus (3)	4/6 (66.67%)	0%	0%	NR	All 6 returned to baseline	0%	6 (100%)
Lim et al. [15]	0 (0%)	Asymptomatic infarcts (2), 1 recurrence	1/6 (16.7%)	1, 16.7%	1/6 (16.7%)	5/6 (83.3%)	6/6 recovered; 1 died later from cancer	2/6 (33.3%)	5 (83.3%)
Ioannidis et al. [9]	0 (0%)	Asymptomatic infarcts (2)	0/10 (0%)	2/10 (20%)	0%	6/10 (60%)	8/10 recovered, 2 died	0%	7 alive patients (100%)
Puri et al. [21]	0 (0%)	Infarcts (4), 1 death	7/7 (100%)	1/7 (14.3%)	0%	0/7	4/7 mRS 0–1; 2/7 mRS 4–5; 1 death	0%	6 alive patients (100%)
Li et al. [14]	0 (0%)	1 recurrence; 1 PICA occlusion (asymptomatic)	1/17 (5.9%)	0%	0%	11/17 (64.7%)	13/17 mRS ≤ 1; 4 with mRS 2–5	1/17 (5.9%)	17 (100%)
Chung et al. [4]	0 (0%)	Fatal hemorrhage (1); PICA occlusion × 2 (no deficits)	7/9 (77.8%)	2/10 (20%)	1/10 (10%)	6/10 (60%)	7/10 mRS ≤ 2; 2 deaths, 1 disabled	0%	8 alive patients (100%)
Lee et al. [13]	0 (0%)	CN VI palsy (resolved in 6 months)	0%	0%	0%	0%	1 (100%)	0%	1 (100%)
Khattar et al. [11]	0 (0%)	0 (0%)	1/1 (100%)	0%	0%	1/1	1/1 (100%) recovery	0%	1 (100%)
Lim et al. [16]	0 (0%)	Mild leg weakness (resolved)	1/1 (100%)	0%	0%	NR	1/1 mRS 0	0%	1 (100%)
Shintoku et al. [24]	0 (0%)	0 (0%)	0% (parent artery occlusion)	0%	0%	NR	1/1 (100%) recovery	0%	1 (100%)
Hiratsuka et al. [8]	0 (0%)	Intracranial HTN (managed)	1/(100%)	0%	0%	NR	1/1 (100%) recovery	0%	1 (100%)

Preservation of PICA patency was achieved in 29 patients (42.6%), although this was lower in surgically treated cohorts. The overall mortality rate across both arms was 10.3% (N = 7), with deaths primarily attributed to rebleeding, infarction, or unrelated systemic causes. Functional recovery, as assessed by the modified Rankin Scale (mRS), showed favorable outcomes in the majority of patients. Among those with available data, 54 (79.4%) had mRS scores < 2 at last follow-up. Finally, long-term radiographic occlusion success was reported in 60 out surviving patients (88.2%).

### Comparison of occlusion rates in surgery vs. endovascular treatment

**Fig. 2:**
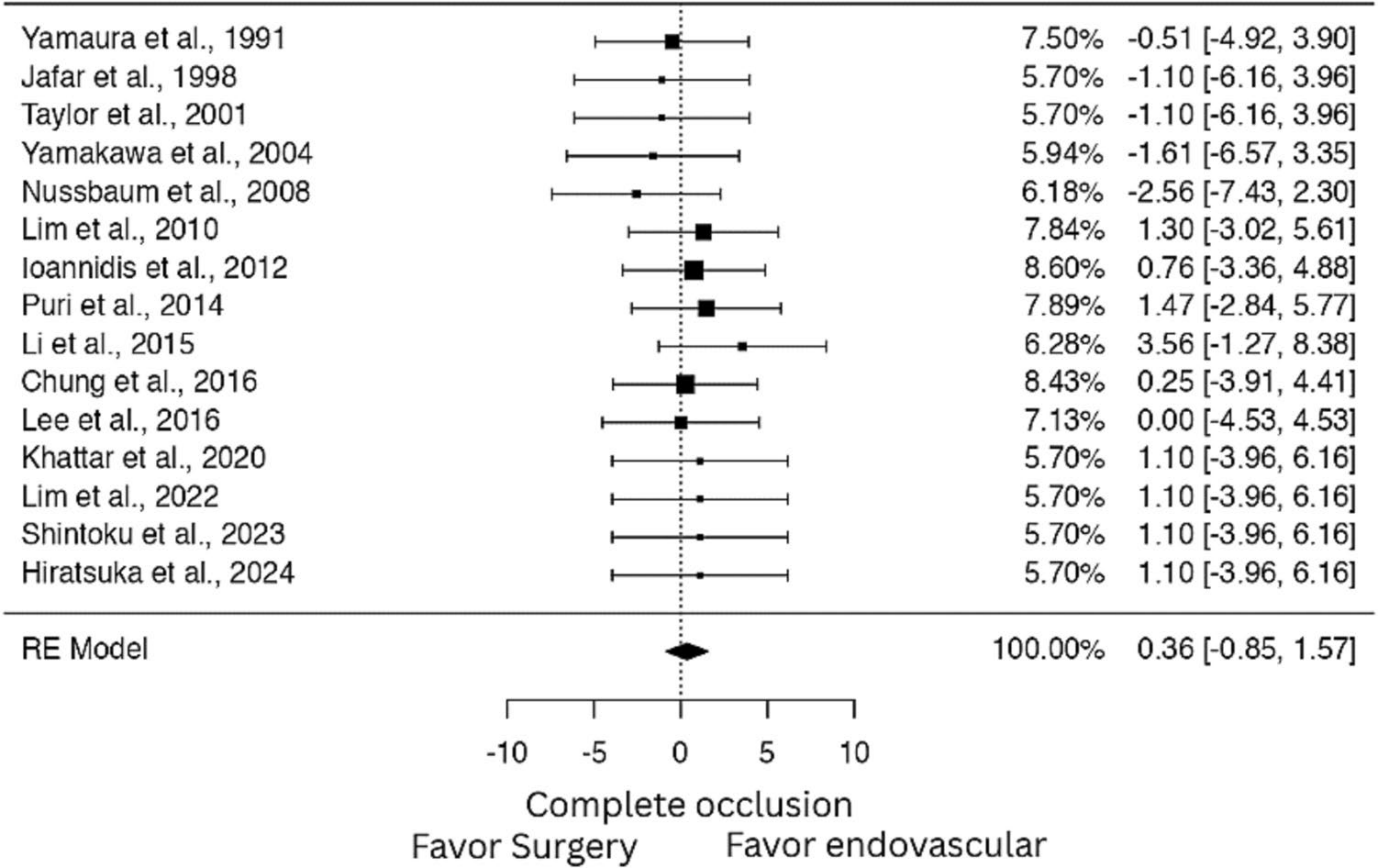
Forest plot comparing complete occlusion rates between surgical and endovascular treatments for distal PICA lesions. No significant difference was observed (OR: 1.43; log OR = 0.360; 95% CI: –0.848 to 1.569) with no observed heterogeneity (I^2^ = 0%)

A meta-analysis of 15 studies showed no significant difference in complete occlusion rates between surgical and endovascular approaches (OR: 1.434; log OR = 0.360; 95% CI: - 0.848 to 1.569; p = 0.981), with no observed heterogeneity (I^2^ = 0%). Publication bias was not detected on the funnel plot or formal testing (Fig. 2).

### One-arm meta-analysis of endovascular treatment outcomes

**Fig. 3:**
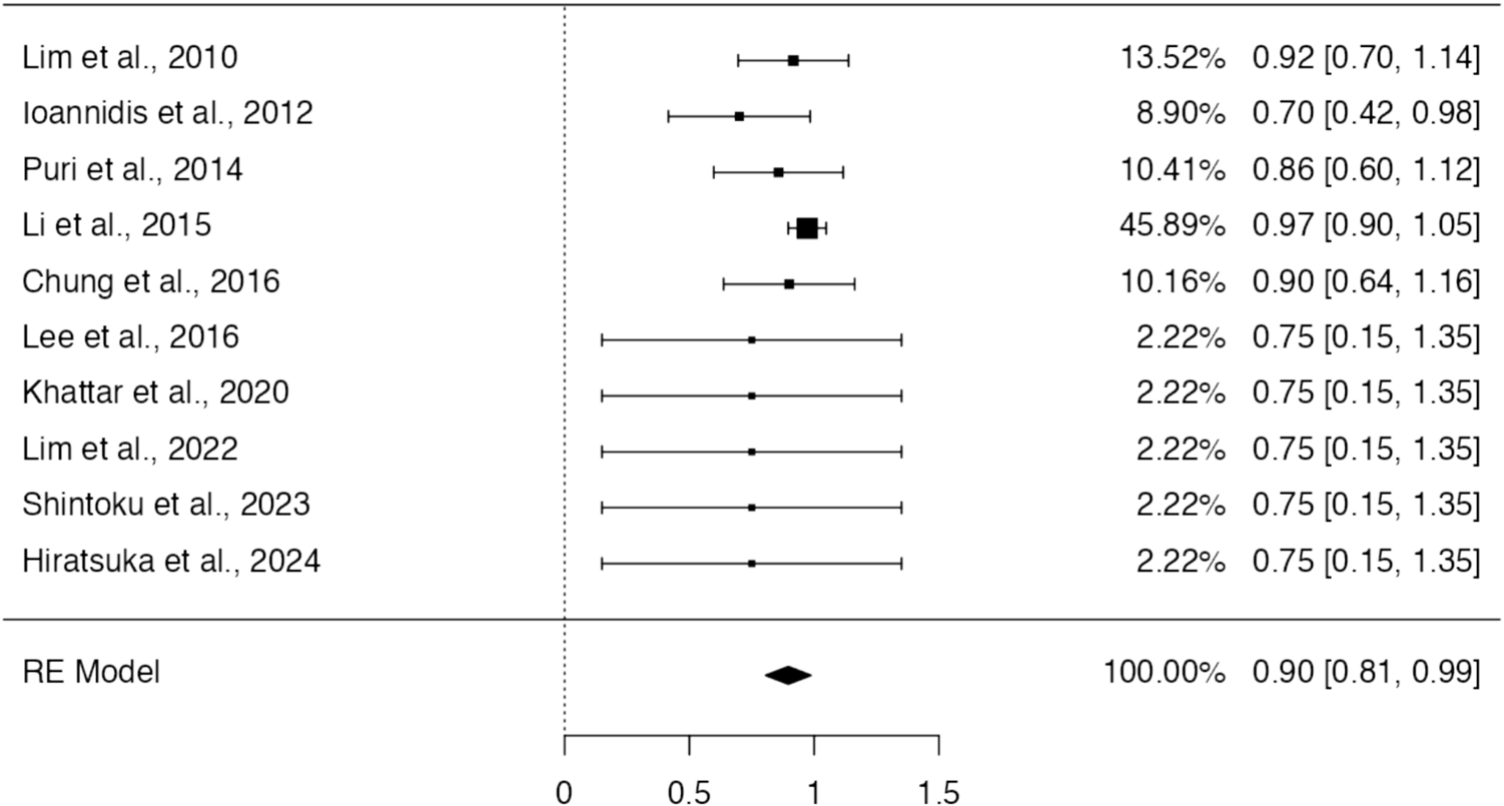
Forest plot showing the pooled complete occlusion rate following endovascular treatment for distal PICA aneurysms (90%; 95% CI: 81–99%)

The complete occlusion rate was 90% (95% CI: 80.6–98.7%, p < 0.001) with low heterogeneity (I^2^ = 14.5%) (Fig. 3). Retreatment was required in 9% of cases (95% CI: 2–15%; p = 0.012), with no observed heterogeneity (I^2^ = 0%). Similarly, the rebleeding rate was 8% (95% CI: 1.9–15.2%, p = 0.012), showing consistent results across studies (I^2^ = 0%). The recurrence rate was also 8% (95% CI: 1.9–15.2%, p = 0.012).

### One-arm meta-analysis of surgical treatment outcomes

The complete occlusion rate was 81% (95% CI: 69.4–94.3%, p < 0.001), with no statistical heterogeneity (I^2^ = 0%), although publication bias was detected (Egger’s p = 0.032). Notably, no cases of retreatment, rebleeding or recurrence were reported across the included studies.

### Comparative evaluation of reconstructive and deconstructive procedures

**Fig. 4:**
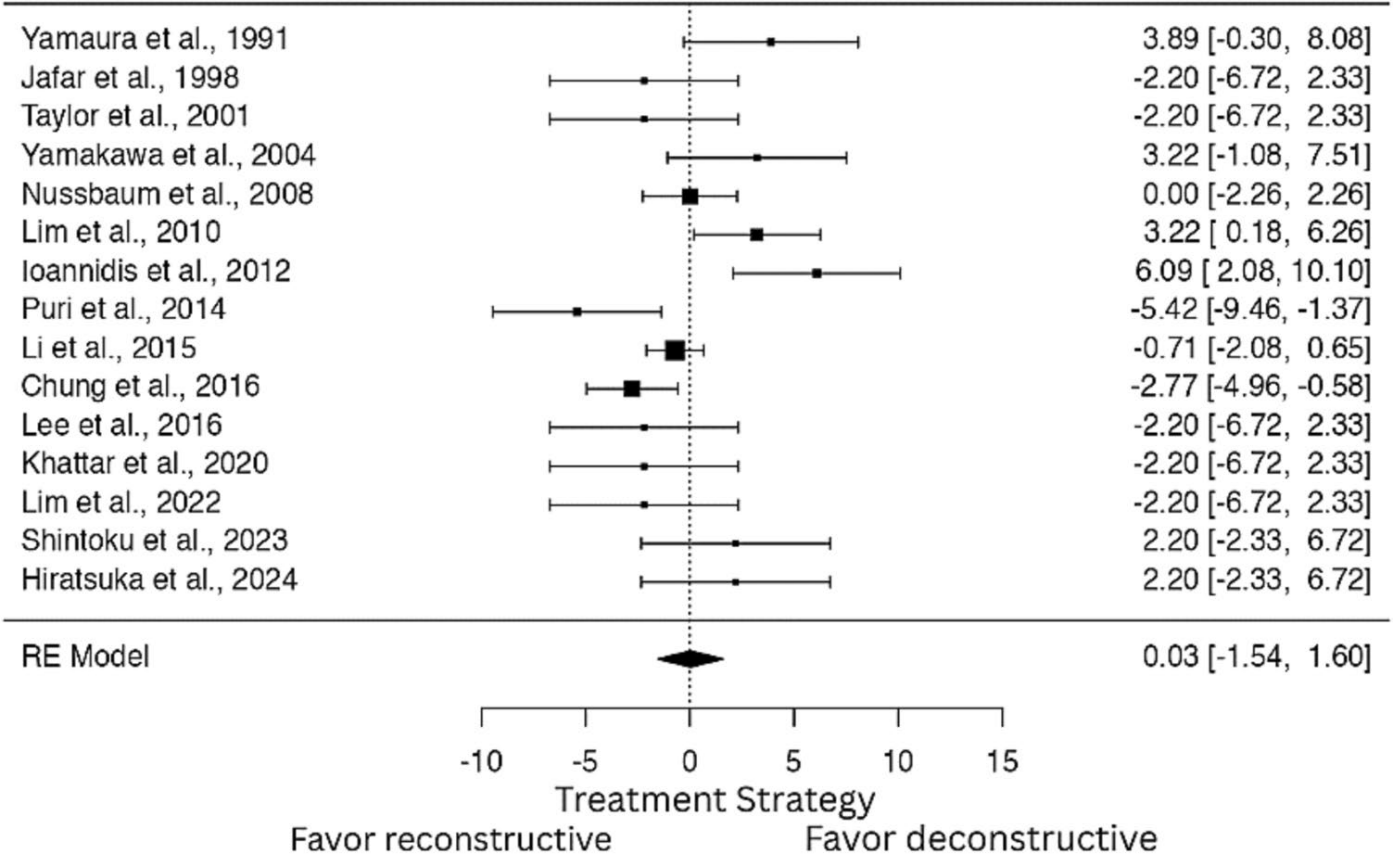
Comparison of Reconstructive vs. Deconstructive Treatment Strategies – Forest Plot of Dichotomous Meta-Analysis. (OR: 0.078; log OR: 0.0287, 95% CI: −1.540 to 1.597, *p* = 0.971, I^2^ = 69%), with moderate heterogeneity (I.^2^ = 69%)

In the pooled one-arm meta-analysis of reconstructive interventions, which included 15 studies, the overall reconstructive methods rate was 45% (95% CI: 0.31–0.67; p < 0.001) (I^2^ = 76.66%). Similarly, the analysis of deconstructive procedures showed a pooled rate of 51% (95% CI: 0.33–0.69; p < 0.001) (I^2^ = 76.66%). To directly compare these two strategies, a dichotomous meta-analysis was conducted. The results indicated no statistically significant difference between reconstructive and deconstructive approaches (OR: 0.078; log OR: 0.0287, 95% CI: −1.540 to 1.597, p = 0.971, I^2^ = 69%) (Fig. 4).

### Proximal tear coverage in deconstructive endovascular procedures

**Fig. 5:**
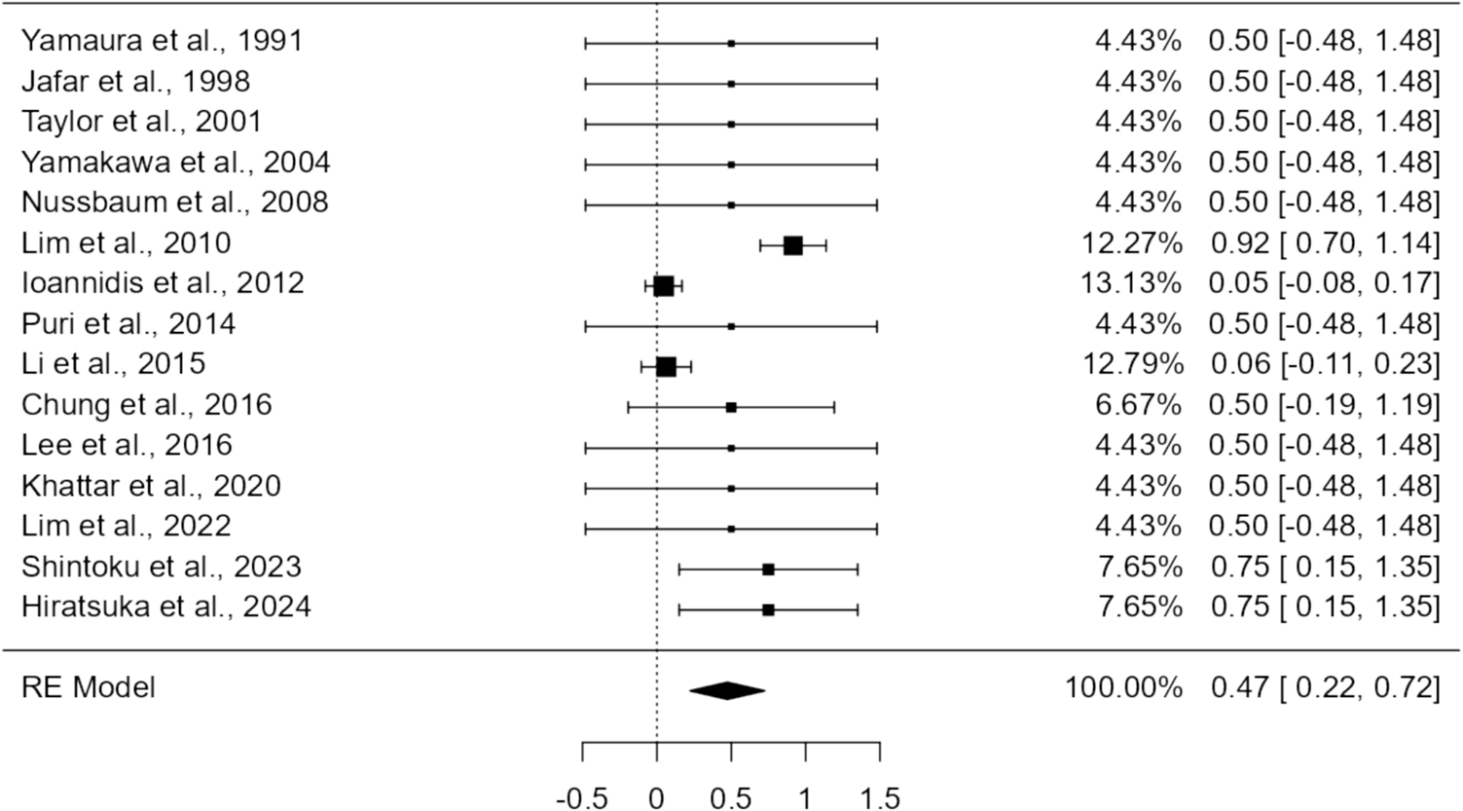
Pooled Proportion of Proximal Tear Coverage in Deconstructive Endovascular Procedures (47%; 95% CI: 0.22–0.72; *p* < 0.001; I.^2^ = 76.02%)

A one-arm meta-analysis using the DerSimonian-Laird random-effects model was conducted to estimate the pooled rate of proximal tear coverage achieved through deconstructive endovascular procedures (Fig. 5). Across 15 studies, the pooled proportion of complete coverage was 47% (95% CI: 0.22–0.72; p < 0.001; I^2^ = 76.02%), indicating that nearly half of the treated lesions achieved effective occlusion of the proximal tear.

### Stroke complication rate across all treatment arms

**Fig. 6:**
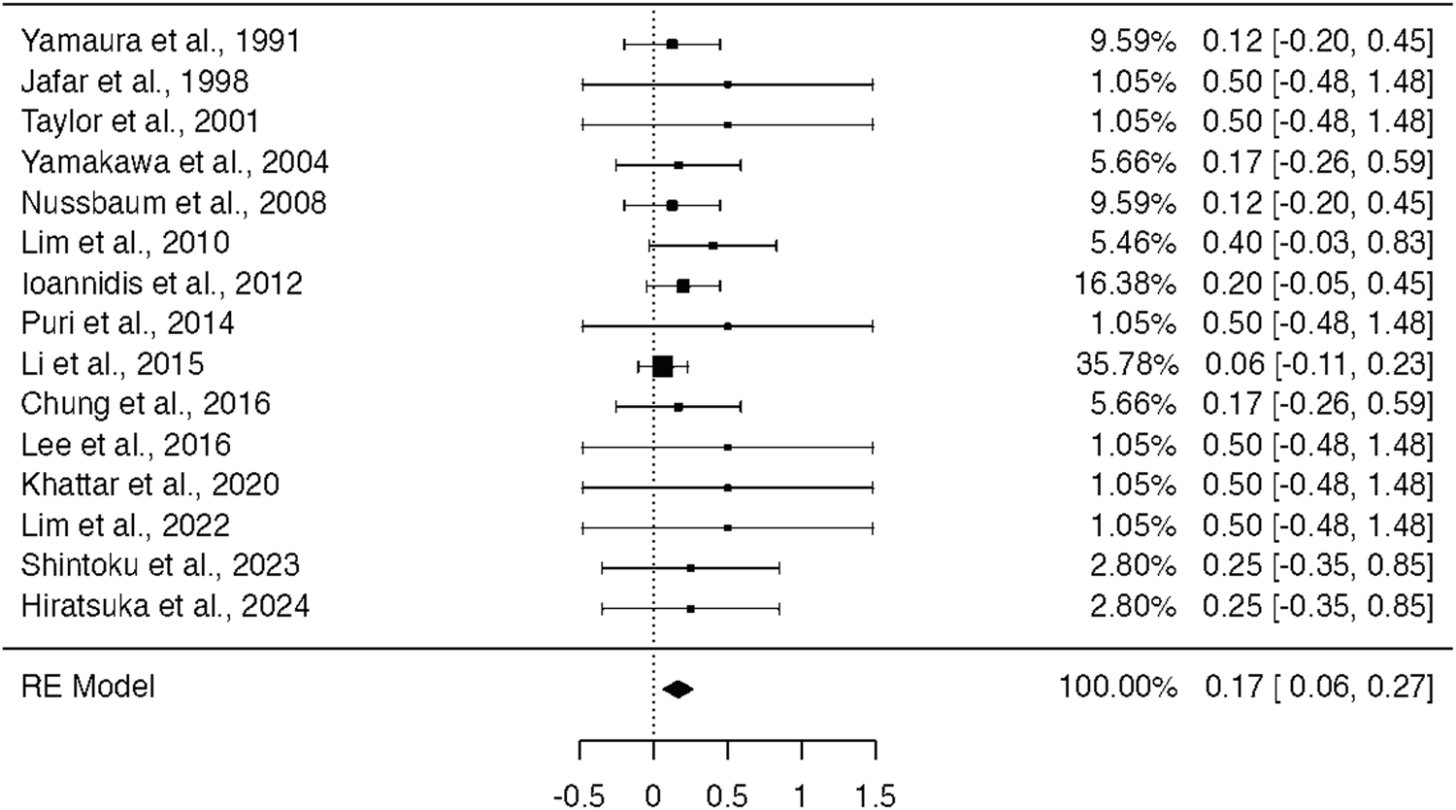
Pooled Stroke Complication Rate Across All Treatment Modalities (17%; 95% CI: 6.5% to 26.6%; *p* = 0.001)

A one-arm meta-analysis evaluating stroke complications across all included studies revealed a pooled event proportion of 16% (95% CI: 6.5% to 26.6%; p = 0.001), indicating a statistically significant complication rate. The analysis demonstrated no heterogeneity (I^2^ = 0%) (Fig. 6).

### Quality assessment

The overall methodological quality of the included studies was acceptable, with most demonstrating low to moderate risk of bias based on standardized assessment tools (Tables 8, 9).

**Table 8:** Quality assessment of case reports using CARE guidelines

Author(s)	Patient Information	Clinical Findings	Diagnostic Assessment	Therapeutic Interventions	Follow-up Outcomes	Discussion/Conclusions	Overall Quality
Jafar et al. [10]	Moderate Risk	Moderate Risk	Low Risk	Low Risk	Low Risk	Low Risk	Moderate Risk
Taylor et al. [18]	Moderate Risk	Moderate Risk	Low Risk	Low Risk	Low Risk	Low Risk	Moderate Risk
Yamakawa et al. [20]	Low Risk	Low Risk	Low Risk	Low Risk	Low Risk	Low Risk	Low Risk
Lee et al. [13]	Low Risk	Low Risk	Low Risk	Low Risk	Low Risk	Low Risk	Low Risk
Khattar et al. [11]	Moderate Risk	Low Risk	Low Risk	Low Risk	Moderate Risk	Low Risk	Moderate Risk
Lim et al. [16]	Moderate Risk	Low Risk	Low Risk	Low Risk	Moderate Risk	Low Risk	Low Risk
Shintoku et al. [24]	Moderate Risk	Low Risk	Low Risk	Low Risk	Moderate Risk	Low Risk	Low Risk
Hiratsuka et al. [8]	Low Risk	Low Risk	Low Risk	Low Risk	Low Risk	Low Risk	Low Risk

**Table 9:** ROBINS-I assessment of the included studies

Author(s)	Confounding	Selection of Patients	Classification of Interventions	Deviations from Intended Interventions	Missing Data	Measurement of Outcomes	Selection of Reported Results
Yamaura et al. [7]	Moderate Risk	Low Risk	Low Risk	Low Risk	Low Risk	Low Risk	Moderate Risk
Nussbaum et al. [19]	Low Risk	Low Risk	Moderate Risk	Low Risk	Low Risk	Moderate Risk	Moderate Risk
Lim et al. [15]	Low Risk	Low Risk	Moderate Risk	Low Risk	Low Risk	Moderate Risk	Low Risk
Ioannidis et al. [9]	Moderate Risk	Low Risk	Moderate Risk	Low Risk	Low Risk	Low Risk	Moderate Risk
Puri et al. [21]	Low Risk	Low Risk	Moderate Risk	Low Risk	Low Risk	Low Risk	Low Risk
Li et al. [14]	Low Risk	Moderate Risk	Moderate Risk	Moderate Risk	Low Risk	Low Risk	Moderate Risk
Chung et al. [4]	Low Risk	Low Risk	Moderate Risk	Low Risk	Low Risk	Low Risk	Low Risk

## Discussion

The majority (91.2%) presented with rupture, predominantly as SAH. Endovascular treatment was the preferred modality (69.1%), achieving a complete occlusion rate of 90% with low retreatment (9%) and rebleeding (8%) rates. Surgical approaches, used in 23.5% of cases, demonstrated comparable occlusion rates (81%). No significant difference in efficacy was found between surgical and endovascular techniques. Functional outcomes were favorable overall, with 79.4% achieving mRS < 2. Stroke occurred in 16% of patients, and flow diversion was not utilized in any case.

The PICA shows significant anatomical variability in both its origin and distal branching patterns, complicating the diagnosis and management of dissecting and pseudoaneurysmal lesions [17]. These lesions primarily affect the distal PICA territory, as highlighted by their radiographic presence in the telovelotonsillar and cortical segments in most studies considered, including those by Lim et al. [15], Ioannidis et al. [9], and Li et al. [14]. Clinical symptoms align with the PICA neuroanatomy, with headaches (32.4%) being the most common initial complaint, followed by signs of brainstem compression or irritation in the posterior fossa. Instances of rare cranial nerve involvement, such as abducent palsy reported by Lee et al. [13], reinforce the localized yet significant nature of this vascular region. Pathophysiologically, most aneurysms originated spontaneously (95.6%), although there were also cases of traumatic dissections, such as one reported by Lee et al. [13] after blunt trauma and another by Lim et al. [15] in a patient with both subarachnoid and intraventricular hemorrhage. Over 40% of assessable cases showed thrombosis within the aneurysm sac, leading to fluctuations in size and diagnostic delays, as noted by Yamakawa et al. [20] and Chung et al. [4]. The presence of associated vascular anomalies emphasizes the anatomical complexity; for instance, Khattar et al. [11] described a case with a dural arteriovenous fistula, and Yamaura et al. [7] observed an additional vertebral artery aneurysm in the same patient, indicating potential shared hemodynamic or structural vulnerabilities. Together, these observations highlight the necessity for specialized diagnostic and treatment approaches that consider PICA’s segmental anatomy.

The pooled complete occlusion rate was slightly higher in the endovascular group (90%) compared to surgery (81%), though the difference was not statistically significant (OR: 1.43; log OR = 0.360; 95% CI: –0.848 to 1.569, p = 0.559; I^2^ = 0%). Importantly, functional outcomes were favorable in both groups, with the majority of patients achieving mRS scores < 2, underscoring the effectiveness of either approach when properly selected. Reconstructive and deconstructive endovascular strategies were evenly represented in the meta-analysis. The dichotomous comparison revealed no significant difference in outcomes between these two techniques (p = 0.971). Notably, proximal tear coverage was achieved in 47% of deconstructive procedures, suggesting room for improvement in technique selection and planning.

Over the past three decades, endovascular management of distal PICA aneurysms has undergone significant transformation. In the early 1990 s, treatment was largely surgical due to limited catheter technology and lack of microdevices suitable for distal vessel navigation. However, contemporary endovascular approaches now dominate, comprising 69.1% of treatments in our reviewed cohort, and are associated with acceptable long-term complete occlusion rates (90%) and low rebleeding (8%) and retreatment (9%) rates. These advances are underpinned by improvements in microcatheter flexibility, embolic materials, and imaging resolution. Notably, despite progress, flow-diverting technologies remain underutilized due to anatomical limitations in distal PICA, highlighting an area for future innovation [5].

Surgical techniques were more frequently employed in cases with hydrocephalus, mass effect, or failed endovascular access, as demonstrated in the series by Nussbaum et al. [19] and Yamakawa et al. [20]. In these scenarios, adjunctive bypass procedures such as occipital artery to PICA anastomosis were employed in patients to preserve distal flow. While technically demanding, these microsurgical strategies remain valuable in anatomically complex or distal lesions not amenable to coiling or stenting. Flow diversion, despite its transformative role in other locations of intracranial aneurysms, was not utilized in any included study, likely due to the small caliber and distal location of the PICA.

Despite favorable outcomes in this cohort, treatment-related complications are a significant concern in managing PICA dissecting and pseudoaneurysmal lesions. Of the 15 studies, postoperative complications occurred in 24 patients (35.3%), with infarction and recurrence as the most common events. A pooled analysis indicated a stroke complication rate of 16% (95% CI: 6.5–26.6%), showing consistency across studies regarding this risk. Asymptomatic infarcts were frequently noted after endovascular PVO, as reported by Lim et al. [15] and Ioannidis et al. [9]. These often indicated distal ischemia due to vessel sacrifice, although most did not lead to permanent deficits. Rebleeding and recurrence were rare but more common in surgically treated lesions. This may be linked to the more fragile morphology of aneurysms selected for microsurgical resection and challenges in achieving durable exclusion of dissected segments. Cranial nerve deficits were very uncommon, with only one instance of abducent palsy reported by Lee et al. [13], resolving spontaneously within six months. Likewise, isolated motor deficits, like mild leg weakness noted by Lim et al. [16], were transient and non-disabling. Notably, no radiographic or symptomatic cases of vasospasm were documented, and access site complications were almost nonexistent, reinforcing the technical safety of current endovascular methods.

Among 68 patients included in this review, complete radiographic occlusion was achieved in 88.2% of survivors, illustrating the durable nature of treatment in this anatomically challenging location. Functional outcomes were similarly encouraging. Of the 68 patients, 54 (79.4%) achieved a mRS score < 2 at last follow-up, indicating a return to functional independence in the majority of cases. Durability varied slightly by treatment modality. These findings were consistent with the series by Ioannidis et al. [9] and Li et al. [14], where endovascular strategies led to stable occlusion and favorable neurological outcomes over follow-up periods ranging from 6 to 30 months. Mortality was reported in 7 patients (10.3%), most commonly due to rebleeding or delayed ischemia, with higher rates observed in the surgical cohorts, such as those by Yamakawa et al. [20] and Chung et al. [4]. Nevertheless, when excluding early deaths, the rate of long-term recovery remained high, with nearly all survivors achieving either full or near-full neurologic restoration. Even in complex cases, such as the traumatic lesion treated by Lee et al. [13] or the double-origin PICA aneurysm described by Hiratsuka et al. [8], successful long-term outcomes were documented without major deficits.

## Limitations, research gaps, and future directions

This review is limited by the small sample size and reliance on case reports and retrospective series, introducing selection and publication bias. Functional and radiographic outcomes were inconsistently reported, with limited data on aneurysm morphology, segmental location, and follow-up duration, which hindered subgroup analysis. Additionally, long-term durability and quality-of-life outcomes remain underexplored. Flow-diverting devices were not used in any case, likely due to anatomical constraints, though newer low-profile stents may warrant investigation. Future multicenter prospective studies are needed to standardize treatment strategies, clarify indications for bypass or reconstruction, and assess long-term neurological and cognitive outcomes.

## Conclusion

This systematic review offers the most comprehensive synthesis to date of dissecting and pseudoaneurysmal lesions of the PICA, a rare and anatomically intricate pathology. Our analysis shows that both endovascular and surgical approaches can achieve durable outcomes, though each carries distinct risk profiles. The absence of flow diversion use highlights a critical gap in current device applicability for distal cerebellar circulation. By consolidating previously fragmented data and presenting pooled outcomes, this study provides new insight into treatment durability and emphasizes the need for individualized, anatomy-specific management strategies in this high-risk patient group.

## Data Availability

No datasets were generated or analysed during the current study.
